# Differential activation of human T cells to allogeneic endothelial cells, epithelial cells and fibroblasts *in vitro*

**DOI:** 10.1186/2047-1440-1-4

**Published:** 2012-04-24

**Authors:** Dmitry Samsonov, Christopher Geehan, Craig B Woda, David M Briscoe

**Affiliations:** 1 The Transplantation Research Center, Division of Nephrology, Department of Medicine, Childrens Hospital Boston, Boston, MA, USA; 2 Department of Pediatrics, Harvard Medical School, Boston, MA, USA; 3 Division of Nephrology, Childrens Hospital Boston, 300 Longwood Ave, Boston, MA, 02115, USA

**Keywords:** Allorecognition, monocytes, APC, Endothelial cells, Fibroblasts, Tubular epithelial cells, T cells

## Abstract

**Background:**

In the direct pathway, T cells recognize intact donor major histocompatability complexes and allogeneic peptide on the surface of donor antigen presenting cells (APCs). Indirect allorecognition results from the recognition of processed alloantigen by self MHC complexes on self APCs. In this study, we wished to evaluate the relative contribution of different intragraft cells to the alloactivation of nave and memory T cells though the direct and the indirect pathway of allorecognition.

**Methods:**

The processing of membrane fragments from IFN-treated single donor endothelial cells (EC), fibroblasts or renal epithelial cells (RPTEC) was evaluated by DiOC labeling of each cell type and flow cytometry following interaction with PBMC. Direct pathway activation of nave CD45RA^+^ or memory CD45RO^+^ CD4^+^ T cells was evaluated following coculture with IFN-treated and MHC class II-expressing EC, fibroblasts or RPTEC. Indirect pathway activation was assessed using CD45RA^+^ or CD45RO^+^ CD4^+^ T cells cocultured with autologous irradiated APCs in the absence or presence of sonicates derived from IFN-treated allogeneic EC, fibroblasts or RPTEC. Activation of T cells was assessed by [^3^H]thymidine incorporation and by ELISpot assays.

**Results:**

We find that CD14^+^ APCs readily acquire membrane fragments from fibroblasts and RPTEC, but fail to acquire membrane fragments from intact EC. However, APCs process membranes from EC undergoing apoptosis.There was a notable direct pathway alloproliferative response of CD45RO^+^ CD4^+^ T cells to IFN-treated EC, but not to fibroblasts or RPTEC. Also, there was a minimal direct pathway response of CD45RA^+^ CD4^+^ T cells to all cell types. In contrast, we found that both CD45RA^+^ and CD45RO^+^ CD4^+^ T cells proliferated following coculture with autologous APCs in the presence of sonicates derived from IFN-treated EC, fibroblasts or RPTEC. By ELISpot, we found that these T cells stimulated via the indirect pathway also produced the cytokines IFN, IL-2, IL-4 and IL-5.

**Conclusions:**

Recipient APCs may readily process membrane fragments from allogeneic intragraft cells, but not from EC unless they are undergoing apoptosis. This processing is sufficient for indirect pathway alloactivation of both CD45RA^+^ and CD45RO^+^ CD4^+^ T cells. Only graft vascular EC mediate direct pathway reactivation of CD4^+^ T cells.

## Background

Recipient CD4^+^ T cell recognition of alloantigen is the central and primary event that ultimately leads to the initiation of allograft rejection [1-3]. It is now well established that alloactivation of T cells occurs via two distinct pathways [4-10]. In the direct pathway of allorecognition, CD4^+^ T cells recognize intact allogeneic major histocompatibility complex (MHC) antigens expressed on the surface of donor cells. In the indirect pathway of allorecognition, CD4^+^ T cells recognize allogeneic peptides processed and presented by self-MHC molecules on the surface of recipient antigen presenting cells (APCs). While the central determinant of alloresponsiveness is recognition of alloantigen, it has become evident that both pathways can contribute to the development of acute and chronic rejection and/or ongoing injury to the graft [7,11,12].

In traditional immunological models, it is suggested that both nave and memory CD4^+^ T cells contribute to rejection through the direct pathway, whereas indirect pathway allorecognition is dominantly associated with activation of the nave repertoire of T cells [7,10,13,14]. This model is based on the hypothesis that the memory T cell repertoire results from expanded populations of T cells that have responded to previous antigen encounters. In contrast, activation of nave populations of T cells is continuous and results from ongoing APC-dependent presentation of alloantigen via both direct and indirect pathways [13]. Since, nave T cells are largely made up of single cells with different specificities, this indicates that the allogeneic response(s) can be very diverse. In addition, it is well known that the precursor frequency of T cells with direct allospecificity is high [4,15]. This suggests that the memory subset alone is unlikely to account for the direct allorecognition response. While the frequency of T cells with indirect specificity is low, they are well established to increase over time following transplantation [16-18]. Thus, it is likely that the continuous activation of nave T cells via indirect presentation of alloantigen by self-APCs will be dominant for the generation of persistent allogeneic responses.

The vascular endothelium functions in the recruitment of recipient immune competent cells into the graft [19-21]. Donor graft vascular endothelial cells (EC) also express MHC class I and II molecules, and have been reported to be potent to provide costimulation for the effective alloactivation of human T cells [20,22-24]. In addition, following re-endothelialization of donor grafts, recipient EC lining vessels within the graft have been found to be efficient in the activation of T cells in a self-restricted manner via the indirect pathway of alloactivation [25,26]. Since both acute and chronic rejection requires interactions among T cells and graft vascular EC, it is proposed that this cell type may serve as a primary candidate to foster the reactivation of T cells, and perhaps the modification of activation responses via both direct and indirect allorecognition [8,25-27]. However, some reports have suggested that endothelial cells lack the ability to provide effective costimulation to T cells [28,29], and others suggest that the expression of MHC class II expression by endothelial cells is not necessary for the rejection response [30]. Nevertheless, these observations do not exclude the importance of donor EC in the promotion of indirect allorecognition via interactions with APCs [23], and their effect in this latter response is poorly understood.

Few studies have evaluated the ability of interstitial intragraft cells to facilitate direct and indirect allorecognition. The lack of expression of costimulatory molecules by fibroblasts and renal tubular epithelial cells (RPTEC) limits their ability to induce T cell activation [31-34]. In this report, we used well-established in vitro models to compare the effect of EC, fibroblasts and RPTEC in direct and indirect alloactivation of nave CD45RA^+^ and memory CD45RO^+^ CD4^+^ T cells. Our findings indicate that EC, but not fibroblasts or RPTEC, provide direct pathway allorecognition to CD45RO^+^ memory subsets of CD4^+^ T cells. In addition, we find that all cell types facilitate indirect allorecognition to both CD45RA^+^ nave and CD45RO^+^ memory CD4^+^ T cell subsets.

## Methods

### Cell Isolation and Culture

Single donor endothelial cells (EC) were isolated from human umbilical cords as previously described [35], and were grown in M199 medium (Bio Whittaker) containing 20% FCS (GIBCO BRL, Grand Island NY) or 10% human serum, EC growth supplement, 1% penicillin/streptomycin, l-glutamine, and heparin. Single donor fibroblasts and renal tubular epithelial cells (RPTEC) were purchased from Clonetics and cultured in CC-4126 FGM 2 or CC-4127 REGM (Clonetics, Walkersville MD) according to the manufacturers protocol. EC, fibroblasts and RPTEC were treated with IFN (1000U/ml, R&D Systems, Minneapolis, MN) for 72h prior to use. EC were used in subculture 34. Apoptosis was induced in EC monolayers following treatment with TNF- (200 U/ml, Biogen, Cambridge MA) and cyclohexamide (2ng/ml, Sigma, St. Louis, MO) for 7h. Peripheral blood mononuclear cells (PBMC) were isolated by Ficoll-Hypaque gradient centrifugation from healthy volunteers. CD4^+^ T cells were isolated from PBMC by positive selection using anti-CD4-coated magnetic beads (Invitrogen, Grand Island NY) according to the manufacturers protocol. CD4^+^CD45RA^+^ and CD4^+^CD45RO^+^ cells were isolated from CD4^+^ cells by negative selection using magnetic beads coated with mouse anti-human CD45RO and CD45RA antibodies (Invitrogen, Grand Island NY).

### Lymphocyte transmigration assays

Transmigration experiments were conducted using EC, fibroblasts or RPTEC monolayers cultured on fibronectin (50 g/ml) coated 3m pore size transwell inserts (Costar, Cambridge, MA, USA) as previously described [35,36]. A total of 310^3^ EC, fibroblasts or RPTEC were seeded onto inserts and were allowed to grow for 45days. Monolayers were treated with IFN (1000U/ml, R&D systems) for the last 72 culture. Confluence of the monolayers was confirmed by Coomasie staining using standard techniques [37]. Monolayers were labeled with DiOC-16 (5 g/ml, Molecular Probes, Eugene OR, USA), which incorporates into the membranes of cells and has an emission at 501nm. A total of 1x10^6^ PBMC were placed into the upper transwell and cells were collected from the bottom well after 1.5h. Following collection, DiOC uptake was analyzed on CD14^+^ CD4^+^ and CD8^+^ cells by flow cytometry.

### Flow cytometry

Confluent monolayers were harvested in Trypsin/EDTA (Sigma, St. Louis, MO) and were analyzed by indirect immunofluorescence staining and flow cytometry as previously described [23]. Briefly, cells were incubated in optimal concentrations of the primary antibodies anti-HLA DR (LB3.1, ATCC, Manassas, VA), negative control mouse IgG (K16/16, a gift from Michael Gimbrone, Brigham and Womens Hospital) or positive control anti-ICAM-1 (RR1/1, a gift from TS Springer) for 30 mins on ice. Cells were washed and subsequently incubated in FITC-conjugated anti-mouse secondary antibody (Jackson, Immunoresearch, West Grove, PA) for an additional 30 mins on ice, and stained cells were washed and fixed in 1% paraformaldehyde prior to analysis. Leukocytes were stained using Phycoerythrin (PE) conjugated anti-human -CD14, -CD4 and -CD8 antibodies (PharMingen, San Diego CA) or isotype negative controls using standard techniques. All stained cells were analyzed using a FACSCalibur cell sorter (Becton Dickinson, Mountain View, CA) and CellQuest and FlowJo software.

### PBMC-allogeneic cell coculture

EC, fibroblasts or RPTEC were cultured to confluence in fibronectin-coated 24-well flat-bottom plates (Costar) coated with fibronectin (50 g/ml) and treated with IFN (1000U/ml) for 72h. PBMC (1x10^6^/well) were added and were cultured for 7days in standard RPMI 1640 medium containing 10% autologous serum. Subsequently, PBMC were harvested by washing with HBSS and CD4^+^ T cells were isolated by positive selection (Invitrogen, Grand Island NY) and were rested in culture medium containing 2.5% of human T-stim without PHA (Becton Dickinson Labware, Bedford, MA) for 5days. The allogeneic HLA mismatch between PBMCs and stimulator EC, fibroblasts or RPTEC was unknown, but was presumed based on consistency of the response in multiple repeated experiments.

### Preparation of Sonicates

Cell membranes were prepared as sonicates from IFN- treated EC, fibroblasts and RPTEC. Exactly 8 10^6^ of each cell type was washed and suspended in 1ml of sterile PBS and was sonicated using a tip sonicator (Branson Sonifer 250) fitted with a 2mm probe. Disrupted cells were centrifuged for 10min at 500g to remove debris. A total of 20l of sonicate was used in each assay. As a quality control, the protein content per ml was measured in occasional sonicate supernatants by standard Bradford assay. Sonicates were frozen at 20C and thawed at 37C before use.

### Indirect allorecognition assays

CD4^+^ T cells (1x10^5^/well) isolated from primary cultures (above) were used with autologous APCs in proliferation assays. Autologous APCs (1x10^5^/well) were isolated from T cell-depleted PBMC, were irradiated (1750rad) and used as stimulators. Assays were performed in round-bottom 96-well plates (Costar). Sonicates from the allogeneic cells, were added (as above) to each culture and, after 6days proliferation was assessed by standard [^3^H]thymidine incorporation assays (1Ci/well) for the last 16h of the coculture.

### ELISPOT assays

ELISpot was performed in 96-well plates (Cellular Technology Ltd., Cleveland, OH) coated overnight with capture cytokine antibodies diluted in sterile PBS. Antibodies were mouse anti-human IFN (clone 2G1, Endogen, Wolburn, MA, 4g/ml), mouse anti-human IL-2 (clone 5355, R&D Systems Inc., Minneapolis, MN, 5g/ml), mouse anti-human IL-4 (clone 8D4-8, BD PharMingen, San Diego, CA, 5g/ml) or rat anti-human IL-5 (clone TRFK5, PharMingen, San Diego, CA, 5 g/ml). After blocking for 1h with PBS/1%BSA, the plates were washed and CD4^+^ T cells (2x10^5^/well) were cultured with autologous APCs (2x10^5^/well) in the absence or presence of sonicate. The plates were incubated at 37C in 5% CO_2_ for 24-36h. Detection antibodies were biotinylated mouse anti-human IFN- (clone B133.5, Endogen, 2g/ml), mouse anti-human IL-2 (clone 5334, R&D Systems Inc., 50ng/ml), rat anti-human IL-4 (clone MP4-25D2, BD PharMingen, 2g/ml) and rat anti-human IL-5 (clone JES1-5A10, PharMingen, 2g/ml). After overnight incubation at 4C, the plates were washed and cytokines were detected using streptavidin HRP (Daco, Carpenteria, CA) and 3-amino-9-ethyl carbazole (Pierce Chemical Co., Rockford, IL). Spots were counted using a computer-assisted ELISpot image analyzer (Cellular Technologies Limited).

### Statistical analyses

Statistical analysis was performed using the student *t* test for two groups of data and by one-way ANOVA for three or more groups. *P* values <0.05 were considered statistically significant.

## Results

### CD14^+^ monocytes acquire membrane fragments from fibroblasts and RPTEC, but not EC

**Figure 1:**
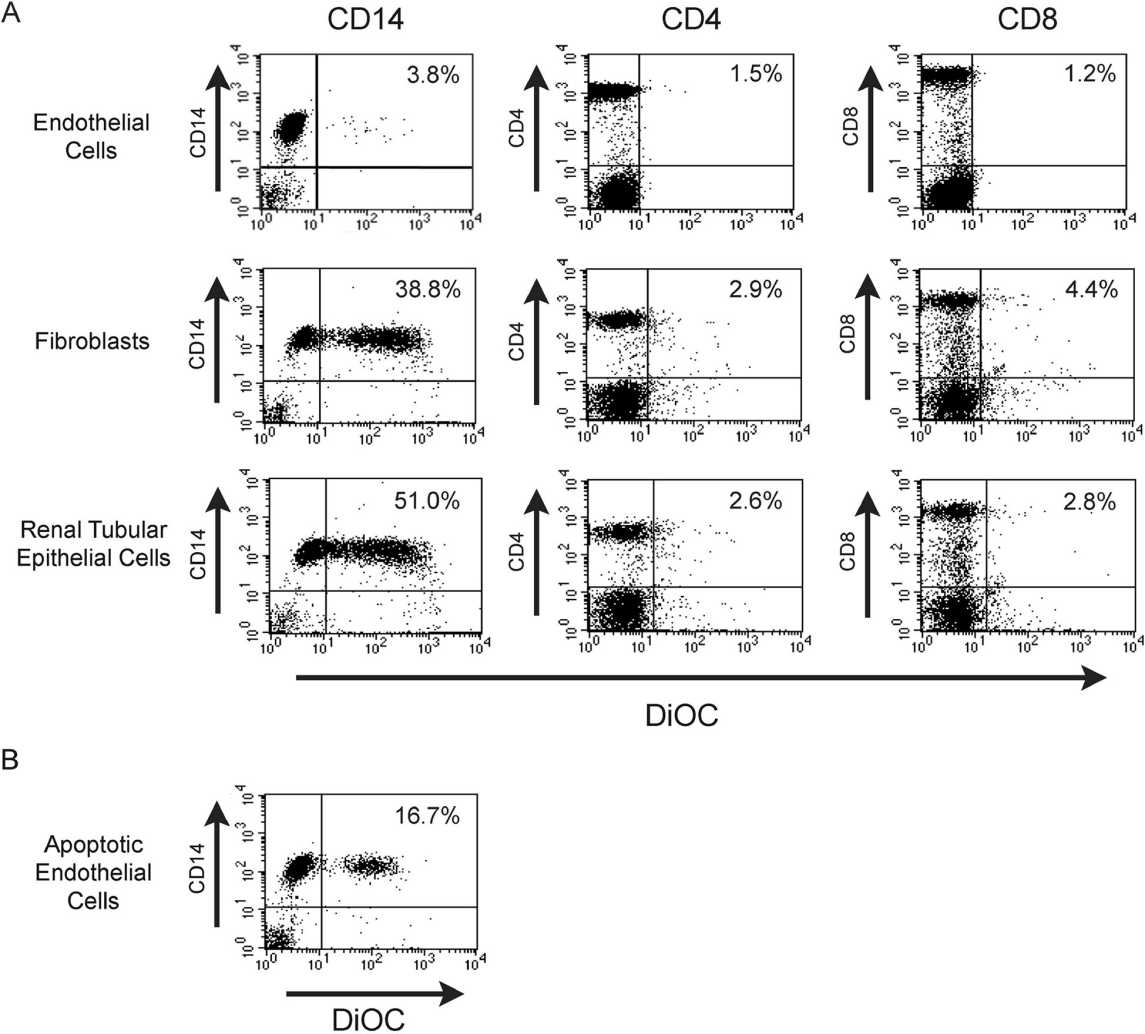
**Transfer of the dye from EC, fibroblasts or RPTEC to CD14**^**+**^**monocytes in transmigration assays.** Confluent monolayers of EC, fibroblasts and RPTEC were grown on transwell inserts and labeled with the lipophylic dye, DiOC-16. Labeled cells were washed extensively before each experiment. A total of 1x10^6^ PBMC were placed into the upper chamber of the transwell, were allowed to transmigrate into the lower chamber and were collected after 1.5h. *Panel A*, DiOC uptake was assessed by FACS analysis on CD14^+^ monocytes and on CD4^+^ or CD8^+^ T cells following transmigration. *Panel B*, Uptake of DiOC was assessed on CD14^+^ monocytes following interactions with EC undergoing apoptosis. Apoptosis was induced in EC by TNF- and cyclohexamide treatment. The percent double positive cells is shown in the right upper corner of each panel. Results are representative of 10 experiments for each cell type, and was identical when different PBMCs and cell lines were used

We initially evaluated whether APCs acquire membrane fragments from allogeneic cells during brief interactions in the course of transmigration. We used a standard transwell model in which PBMC were allowed to transmigrate through confluent IFN-treated EC, fibroblasts or RPTEC. Prior to the assay, cells were labeled with lipophylic DiOC-16, which is well established to stably incorporate into cell membranes. As illustrated in Figure 1, we found that 3565% of CD14^+^ monocytes acquired dye after interaction with both fibroblasts and RPTEC. However, surprisingly, the transfer of dye was very limited after interaction with EC. We also found that neither CD4^+^ T cells nor CD8^+^ T cells acquire dye from any allogeneic cell type indicating that the transfer was related to phagocytosis of membrane rather than through cell surface membrane transfer (as can occur in the semi-direct pathway of allorecognition [7,38]). To further confirm that intact EC fail to transfer membrane to APCs, we also assessed transfer when PBMC transmigrated across EC undergoing apoptosis (TNF- and cyclohexamide- treated cells). As illustrated in Figure 1B, we find that APCs acquire DiOC-labeled membrane from apoptotic EC (15-25% cells) as compared to untreated or IFN-treated EC (3-10% cells). In contrast, the transmigration of PBMC across apoptotic fibroblasts or RPTEC did not alter DiOC-labeled membrane uptake from that described above (data not shown). Therefore, it is possible that acute injury or alloimmune targeting of EC may be a factor in the initiation of indirect processing of alloantigen by APCs. This process may result in crosstalk between both pathways of allorecognition, as described [8].

### Direct and indirect allorecognition by CD45RA^+^ and CD45RO^+^ CD4^+^ T cells in response to IFN-treated EC, fibroblasts or RPTEC

**Figure 2:**
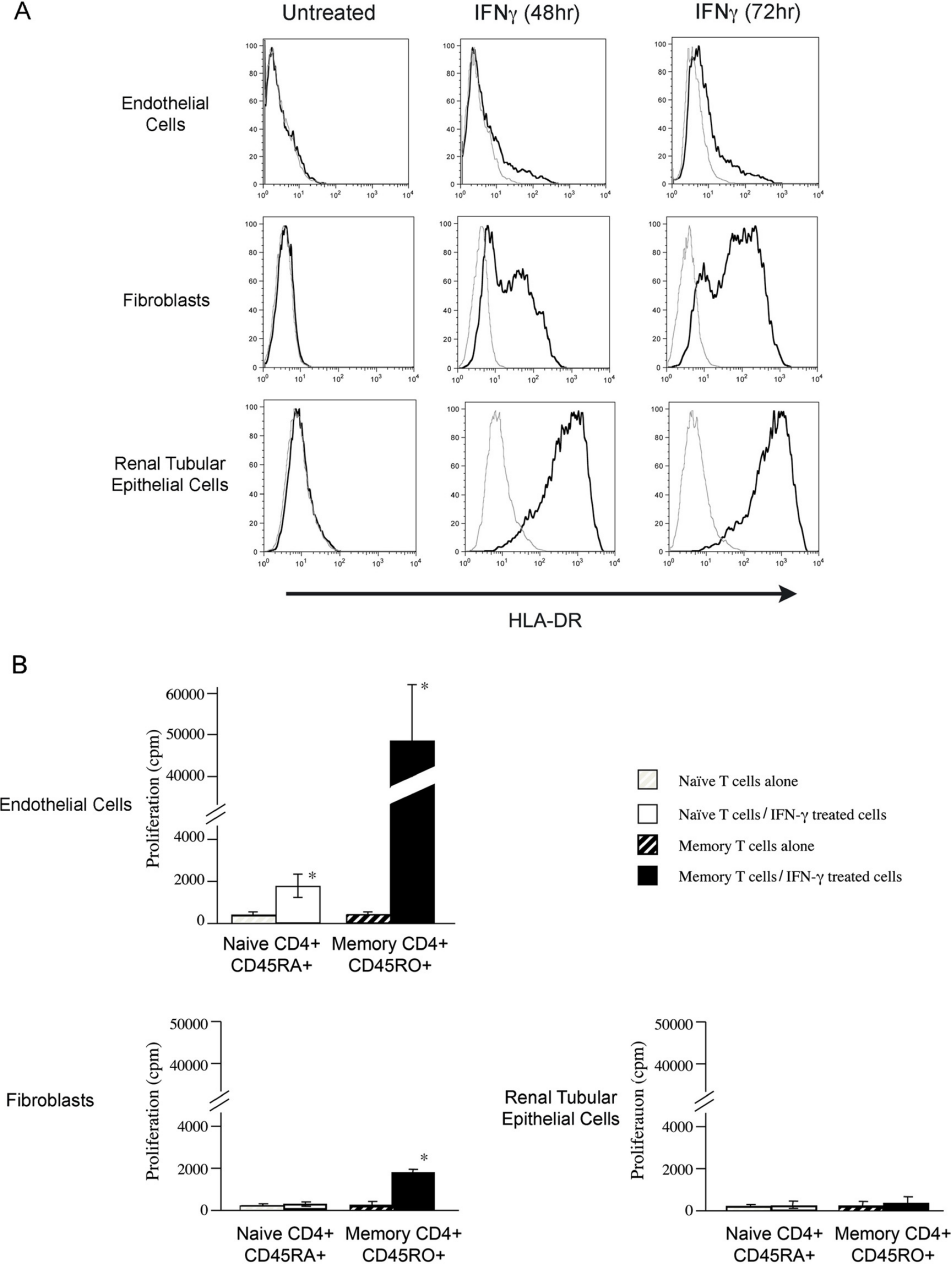
**Direct alloactivation of naive and memory T cells by IFN-treated EC, fibroblasts and RPTEC.***Panel A*, FACS analysis of MHC class II on EC, fibroblasts and RPTEC, either untreated or following treatment with IFN for 48 and 72 h, as indicated, using mouse anti-human HLA-DR (LB3.1, solid black lines) or negative control mouse IgG (K16/16, grey lines). The expression of ICAM-1 served as a positive control (not shown). *Panel B*, Nave CD45RA^+^ and memory CD45RO^+^ CD4^+^ T cells were isolated from PBMC. A total of 1x10^5^ CD45RA^+^CD4^+^ T cells (white bars) or CD45RO^+^CD4^+^ T cells (black bars) were cocultured either alone (striped bars) or with 5x10^4^ allogeneic IFN-treated EC, fibroblasts or RPTEC (solid bars) for 5days. Proliferation was assessed by [^3^H] thymidine incorporation. Results are representative of at least 3 experiments performed in triplicate 1SD (*P<0.05) using cells from different donors and responders

We next wished to compare the ability of EC, fibroblasts and RPTEC to induce direct and indirect pathway alloactivation of nave CD45RA^+^ and memory CD45RO^+^ CD4^+^ T cells. CD45RA^+^ and CD45RO^+^ cells were isolated by negative selection from pure populations of CD4^+^ T cells (>90% purity by FACS, data not shown) and were cocultured with IFN-treated EC, fibroblasts or RPTEC for 5days. As illustrated in Figure 2A, and consistent with other reports [23,39,40], we find that our IFN-treated EC, fibroblasts and RPTEC express high levels of MHC class II. However, CD45RO^+^CD4^+^ T cells showed significant proliferation only in response to culture with allogeneic EC, and had minimal proliferative responses when cultured with fibroblasts or RPTEC (Figure 2B). In contrast, there was a minimal proliferative response of CD45RA^+^CD4^+^ T cells to all allogeneic stimulator cell types (Figure 2B). This observation is consistent with previously published reports indicating that EC function in the activation of allogeneic T cells [22,23,41,42]. They are also consistent with previous studies indicating that EC fail to provide sufficient costimulation for the initiation of nave T cell activation responses [23,28].

**Figure 3:**
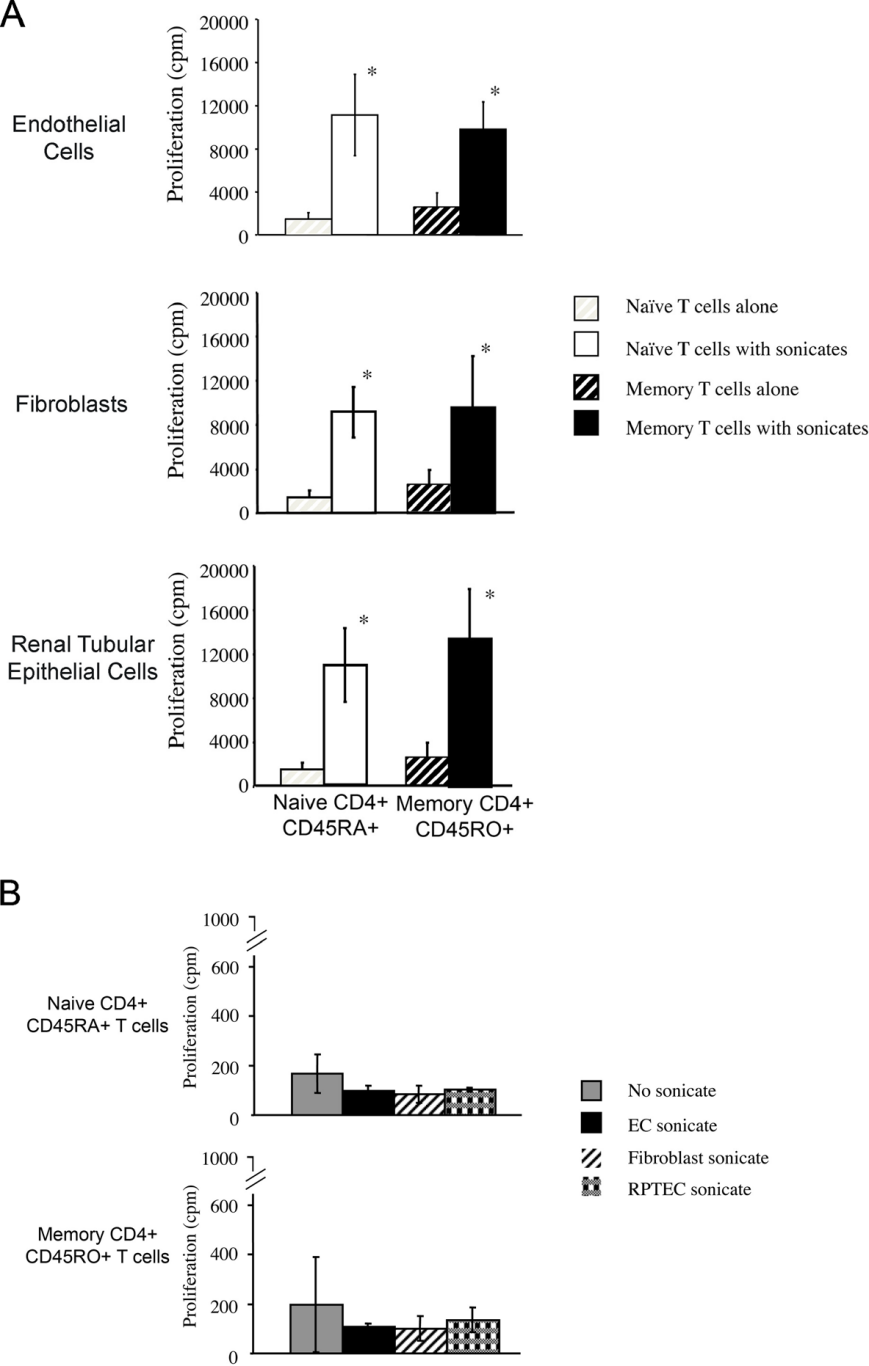
**Indirect alloactivation of naive and memory T cells following coculture with autologous APCs in the presence of sonicates derived from allogeneic EC, fibroblasts or RPTEC.** Nave CD45RA^+^ cells and memory CD45RO^+^ CD4^+^ T cells were isolated from PBMC as described in Methods. *Panel A*, 1x10^5^ CD45RA^+^CD4^+^ T cells (white bars) or CD45RO^+^CD4^+^ T cells (black bars) were cocultured for 6days with 1x10^5^ irradiated autologous PBMC either alone (striped bars) or in the presence of sonicate (solid bars) prepared from allogeneic IFN treated EC, fibroblasts or RPTEC. Proliferation was assessed by [^3^H] thymidine incorporation. Results are representative of 3 different experiments performed in triplicate 1SD (*P<0.05). *Panel B*, 1x10^5^ CD45RA^+^ (Upper Panel) or CD45RO^+^ (Lower Panel) CD4^+^ T cells were cultured for 6days either alone (gray bars) or with sonicate prepared from allogeneic IFN-treated EC (black bars), fibroblasts (striped bars) or RPTEC (checked bars). Proliferation was assessed by [^3^H] thymidine incorporation. The illustrated results in *Panels A and B* are representative of 3 different experiments performed in triplicate 1SD

In order to evaluate the effect of each cell type in indirect alloactivation, we next cocultured CD45RA^+^ or CD45RO^+^ CD4^+^ T cells with autologous APCs in the absence or presence of sonicate derived from allogeneic 72h IFN-treated EC, fibroblasts and RPTEC (as shown in Figure 2A*)*. In these assays, T cells cultured in the presence of autologous APCs without sonicate served as a negative control. Illustrated in Figure 3A, we find that both nave CD45RA^+^ as well as memory CD45RO^+^ CD4^+^ T cells proliferate to sonicates prepared from all cell types. As a control, each T cell subset failed to proliferate to sonicate alone in the absence of autologous APCs (Figure 3B). These observations indicate that indirect allorecognition is a most potent mechanism for the allogeneic activation of both nave and memory T cells.

### Effect of sensitization on indirect allorecognition by CD4^+^ T cells

**Figure 4:**
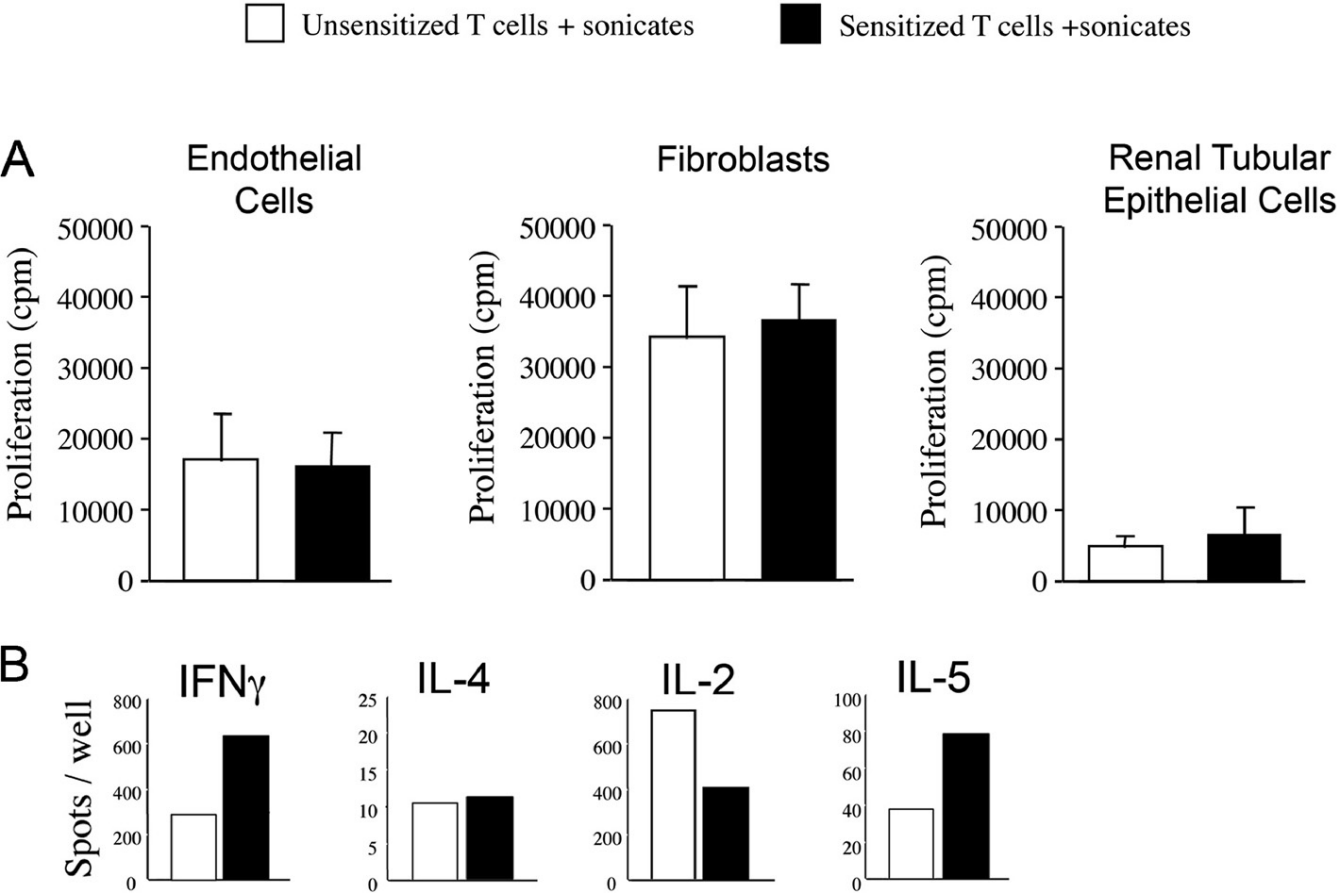
**Indirect alloactivation of unsensitized and sensitized CD4**^**+**^**T cells by autologous APCs in the presence of sonicates derived from allogeneic EC, fibroblasts or RPTEC.** PBMC were cultured either alone on plastic or with confluent monolayers of IFN-treated EC, fibroblasts or RPTEC for 7days. Subsequently, CD4^+^ T cells were isolated from cultures by positive selection and, after a 5day rest period, were used as responders with irradiated autologous APCs in the presence of sonicate. Sonicate was derived from IFN-treated EC, fibroblasts or RPTEC, as indicated, and the identical stimulator cell was used in primary and secondary cultures. *Panel A*, proliferation of unsensitized (white bars) and sensitized (black bars) CD4^+^ T cells as assessed by [^3^H] thymidine incorporation for the last 18h of a 6day culture. Results are representative of at least 3 different experiments performed in triplicate 1SD using different donors of PBMC and different EC, fibroblasts and RPTEC. *Panel B*, representative ELISpot analysis of IFN, IL-4, IL-2 and IL-5 following 48h culture of unsensitized or sensitized CD4^+^ T cells with sonicate derived from allogeneic EC. The results are representative of 3 different experiments. Similar cytokine production was found following indirect alloactivation of T cells by fibroblasts and RPTEC, but the response in general was less pronounced than that found using EC sonicates (not shown)

We next created an in vitro model to evaluate whether there is a difference in indirect responses among unsensitized and sensitized CD4^+^ T cells. For this purpose, we cultured PBMC with IFN-treated EC, fibroblasts or RPTEC. After 7days, the CD4^+^ T cells were isolated from the cultures and were rested in culture medium alone. After 5days of rest, these sensitized T cells (or freshly isolated unsensitized cells) were then used as responders in cocultures with autologous APCs in the absence or presence of sonicates derived from the allogeneic EC, fibroblast or RPTEC used in the primary culture. Proliferation was again assessed on day 6. Illustrated in Figure 4, we found no difference in the proliferative responses of unsensitized or sensitized T cells in these indirect allorecognition assays. By ELISpot (48h), we found variations in individual cytokine production between sensitized and unsensitized cells (Figure 4B). However, the overall production of cytokines was at high levels reflective of the activation status of the T cells and there were no significant differences between responses in unsensitized and sensitized cells (data not shown). ELISpot counts for IFN were generally higher when EC were used as the alloantigen, as compared to assays where fibroblasts and RPTEC were used (Figure 4B). Collectively, these observations further confirm that the indirect response is potent to initiate T cell activation, and indicate that T cells primed through the indirect pathway may be reactivated within allografts upon encounter with self-APCs.

## Discussion

In the current study, we find that MHC class II-expressing EC are potent for direct pathway allogeneic reactivation of memory T cell subsets, and that direct pathway activation does not occur following interactions with fibroblasts or renal tubular epithelial cells. In addition, we find that CD14^+^ monocyte/APCs readily acquire membrane particles from fibroblasts as well as renal epithelial cells and that this process is sufficient to induce indirect pathway allogeneic activation of both nave and memory T cells. In contrast, the ability of monocytes to phagocytose membrane particles from intact EC is limited, and acquisition by APCs requires apoptosis of EC. Nevertheless, once APCs process particulates from allogeneic EC, these APCs are most potent to induce indirect alloactivation of T cell subsets. These observations are consistent with the hypothesis that memory CD4^+^ T cells contribute to rejection through direct pathway interactions with allogeneic EC. In addition, they indicate that indirect pathway allorecognition can occur following processing of alloantigen by all cell types, and this pathway is dominant for the activation of nave repertoires of human T cells.

The indirect pathway of allorecognition was initially described based on the observation that passenger leukocyte-depleted renal allografts were rejected at a slower pace than APC replete allografts [43]. It is suggested that recipient APCs traffic through the graft, take up and process soluble MHC alloantigens, as well as dead or necrotic donor cells. After migrating back to lymph nodes these APCs process and present alloantigens as peptides on self MHC class II molecules to nave CD4^+^ T cells [9,10]. Consistent with this model, our in vitro studies indicate that human nave and memory T cells respond to autologous APCs that have processed membrane particulates from allogeneic cells. In addition, previous studies have confirmed that donor MHC peptides are presented to recipient T cells through the indirect pathway [44,45], and that these responses occur in patients with chronic allograft rejection [11,16,17]. Indeed, in vivo studies have confirmed that MHC-derived antigens from allografts are commonly processed and can initiate T effector responses [7,8,10,13,46,47].

During rejection, endothelial cells function to elicit the recruitment of monocyte/APCs as well as activated T cells into the graft [19,20]. Multiple studies by our own group, as well as others, have determined that this process and interactions with allogeneic EC may facilitate Th1 [22,48], Th2 [21,49], or Th17 [50] activation as an integral component of the inflammatory response. Our studies highlighted in this report further confirm these findings, and indicate that this response typically results from EC-dependent reactivation of the CD45RO^+^ memory T cell subset. In contrast, our observations indicate that interstitial cells are ineffective for direct pathway alloactivation of T cells. These findings are consistent with several other reports [31-34,51] suggesting that fibroblasts and epithelial cells have a limited ability to provide costimulation to T cells. However, our new findings in this report indicate that once APCs process membrane fragments from interstitial cells, they are most potent to initiate indirect pathway alloactivation. These findings lead to the interpretation that antigens derived interstitial cell types are dominant to induce indirect pathway alloactivation.

An intriguing possibility is that the processing of alloantigen derived from interstitial cell types precedes subsequent emergence of APCs from the graft and their differentiation and maturation into mature APCs/dendritic cells. Indeed, several studies have confirmed that processing is a prerequisite for dentritic cell maturation [52]. Thus, another interpretation of our findings is that the inhibition of the recruitment of monocytes into an allograft or the inhibition of alloantigen processing will attenuate the development of nave T cell activation/indirect allorecognition.

A limitation of this study is that there is individual variation in precursor frequencies of alloreactive memory T cells. In the ideal experiment, one might control for precursor cells, and each cell type should be tested against one specific alloantigen that represents a single MHC-derived peptide. Another limitation is that the maturation and differentiation of APCs into mature dendritic cells and their subsequent interaction with nave and memory T cells requires specific inflammatory mediators and perhaps the local lymphoid microenvironment. Nevertheless, our findings indicate that endothelial cells are potent to induce alloresponses through both the direct and the indirect pathway. In contrast, fibroblasts and RPTEC selectively activate T cells through the indirect pathway.

## Conclusions

Overall, these in vitro studies clearly demonstrate that different interstitial cell types have potential to activate allogeneic T cells either through the direct or the indirect pathway of allorecognition. Our studies indicate that memory T cells are reactivated upon encounter with graft vascular EC, and that interstitial cells are weak or inefficient to elicit direct pathway reactivation of this subset. In contrast, we find that the indirect pathway is potent to induce the alloactivation of both nave and memory T cells, and that indirect responses likely occur as a result of APC processing of interstitial cells. Once self-APCs migrate into allografts and have interacted with intragraft cells, their subsequent encounter with T cells (within an allograft or within a lymph node) has potential to mediate indirect alloactivation.

## Competing interests

The authors declare that they have no competing interests.

## Authors contributions

DS performed the studies, analyzed and interpreted data and drafted the manuscript. CG performed some studies and analyzed data. CW performed some studies and analyzed data. DMB conceived and designed the study, participated in the design of and the interpretation of experiments, analyzed and interpreted data, edited and approved the final draft of the manuscript. All authors read and approved the final manuscript.

## Financial conflicts

The authors have no financial conflicts.
